# COVID-19 Outbreak: Pathogenesis, Current Therapies, and Potentials for Future Management

**DOI:** 10.3389/fphar.2020.563478

**Published:** 2020-10-16

**Authors:** Md. Farhad Hossain, Sharifa Hasana, Abdullah Al Mamun, Md. Sahab Uddin, Mir Imam Ibne Wahed, Sabarni Sarker, Tapan Behl, Irfan Ullah, Yesmin Begum, Israt Jahan Bulbul, Md. Shah Amran, Md. Habibur Rahman, May N. Bin-Jumah, Saad Alkahtani, Shaker A. Mousa, Lotfi Aleya, Mohamed M. Abdel-Daim

**Affiliations:** ^1^Department of Physical Therapy, Graduate School of Inje University, Gimhae, South Korea; ^2^Pharmakon Neuroscience Research Network, Dhaka, Bangladesh; ^3^Department of Pharmacy, Southeast University, Dhaka, Bangladesh; ^4^Department of Pharmacy, Faculty of Science, University of Rajshahi, Rajshahi, Bangladesh; ^5^Department of Pharmacy, Faculty of Life and Earth Sciences, Jagannath University, Dhaka, Bangladesh; ^6^Chitkara College of Pharmacy, Chitkara University, Punjab, India; ^7^Kabir Medical College, Gandhara University, Peshawar, Pakistan; ^8^Department of Pharmaceutical Chemistry, University of Dhaka, Dhaka, Bangladesh; ^9^Department of Global Medical Science, Yonsei University, Seoul, South Korea; ^10^Department of Biology, College of Science, Princess Nourah bint Abdulrahman University, Riyadh, Saudi Arabia; ^11^Department of Zoology, College of Science, King Saud University, Riyadh, Saudi Arabia; ^12^Pharmaceutical Research Institute, Albany College of Pharmacy and Health Sciences, New York, NY, United States; ^13^Chrono-Environnement Laboratory, UMR CNRS 6249, Bourgogne Franche-Comté University, Besançon, France; ^14^Pharmacology Department, Faculty of Veterinary Medicine, Suez Canal University, Ismailia, Egypt

**Keywords:** coronavirus, coronavirus disease 2019, severe acute respiratory syndrome coronavirus-2, Middle East respiratory syndrome coronavirus, transmission, therapeutic interventions

## Abstract

At the end of 2019, a novel coronavirus (CoV) was found at the seafood market of Hubei province in Wuhan, China, and this virus was officially named coronavirus diseases 2019 (COVID-19) by World Health Organization (WHO). COVID-19 is mainly characterized by severe acute respiratory syndrome coronavirus-2 (SARS-CoV2) and creates public health concerns as well as significant threats to the economy around the world. Unfortunately, the pathogenesis of COVID-19 is unclear and there is no effective treatment of this newly life-threatening and devastating virus. Therefore, it is crucial to search for alternative methods that alleviate or inhibit the spread of COVID-19. In this review, we try to find out the etiology, epidemiology, symptoms as well as transmissions of this novel virus. We also summarize therapeutic interventions and suggest antiviral treatments, immune-enhancing candidates, general supplements, and CoV specific treatments that control replication and reproduction of SARS-CoV and Middle East respiratory syndrome coronavirus (MERS-CoV).

## Introduction

The name of coronavirus (CoV) comes from corona that shows crown-shaped spikes on the outer surface of the virus and this virus belongs to the Nidovirales order and coronaviridae family (Zhang and Liu, 2020). CoV mainly causes enzootic infections not only in birds but also in mammals including humans demonstrated by recent data (Schoeman and Fielding, 2019). The spread of the novel CoV has faced periodically in 210 countries and territories around the world (WHO, 2020). In humans, CoV increases the severity of common cold to severe respiratory infections, which turn into death. Recently, the Chinese health authorities and Chinese Center for Disease Control identified a novel flu-like CoV that is genetically related to severe acute respiratory syndrome (SARS-CoV) and Middle East respiratory syndrome coronavirus (MERS-CoV) (Chan et al., 2020a; Chen Y. et al., 2020). This novel CoV originated at the end of December 2019 at Wuhan, China where seafood, bats, snacks, dogs, and other animals are regularly sold and bought and the outbreaks of this virus spread out rapidly within a short time resulting in severe illness to death. Thereafter, the World Health Organization (WHO) officially confirmed the disease and named as Coronavirus disease 2019 (COVID-19) (Yao et al., 2020).

In December 2019, the first cases were identified (Du Toit, 2020). Five patients who had acute respiratory distress syndrome (ARDS) were hospitalized from 18 December 2019 to 29 December 2019, and one patient died (Ren et al., 2020). By January 2, 2020, there had been 41 admitted hospital patients, less than half of whom had chronic disorders, including diabetes, asthma, and cardiovascular problems (Huang C. et al., 2020). Some patients were expected to be diagnosed with nosocomial infection in that hospital. It is assumed that COVID-19 is not a very heat-dispersive virus (transmitted from one patient to many others) but instead is likely to spread due to unknown mechanisms in many patients in various hospital locations. Several reports revealed that human transmission of COVID-19 have occurred in various ways such as eyes, nose, and oral routes, and predominantly from the person to person contact through a sneeze, coughing, inhalation, and other means. This report was supported and favored by the fact that the most COVID-19 cases occurred within families as well as among people who did not go to the animal market located in Wuhan, China (Graham Carlos et al., 2020; Wu P. et al., 2020). Additionally, COVID-19 can be transmitted by medical wastes or used personal protective equipment (PPE). So, the mode of transmission is very vital to inhibit the progression of COVID-19. On the other hand, this virus affects not only the lung but also various organs, including the heart, brain, nervous system, and kidney (Zhang J. et al., 2020). In this article, we have summarized various modes of transmission and infected organs by COVID-19. Several therapeutic candidates including immune enhancer or antiviral drugs such as favipiravir (FPV), remdisivir (RDV), lopinavir (LNV), ribavirin (RVN), arbidol (ARB), and antimalarial drugs including chloroquine and hydroxychloroquine have been recommended as effective investigational drugs, some of which are in under clinical trial in animals and humans (Kabir et al., 2020a). In this pandemic situation, at the end of March 2020, chloroquine phosphate and hydroxychloroquine sulfate were permitted by Food and Drug Administration (FDA) as emergency use to treat COVID-19 patients (FDA, 2020). Besides, FPV, the antiviral drug, which was permitted to treat influenza in China and Japan, and now this drug is under clinical trial for treating COVID-19 (Fujifilm Global, 2020).

However, epidemiology and transmission of COVID-19 are unclear, and there is no specific vaccine and treatment yet. The purpose of this review is to elucidate the etiology, epidemiology, symptoms, and transmission according to the recent data. Furthermore, we also focus on the possible therapeutic candidates as a basic treatment for the novel COVID-19.

## Etiology and Epidemiology of COVID-19

### Etiology

The virus is of zoonotic origin and has the potential of causing infection from human to human transmission. There are four genera of CoV namely, α-coronavirus (α-CoV), β-coronavirus (β-CoV), γ-coronavirus (γ-CoV), and δ-coronavirus (δ-CoV) are under the subfamily of Orthocoronavirinae (Wong et al., 2019). Six CoV species including 229E, NL63, OC43, HKU1, SARS-CoV, and MERS-CoV are associated with human disease (Li et al., 2007; Hulswit et al., 2019). All viruses that belong to the group coronaviridae are called CoV. They are (+) single-stranded RNA (ssRNA) viruses dispersed in a wide variety of warm-blooded chordates and are known for several diseases in different organ systems such as the brain, gastrointestinal tract (GIT), lungs, and liver (Zhu et al., 2020). Four virus species, namely, hCoV-229E, OC43, NL63, and KKU1 are widespread and known to affect the respiratory tract (Su et al., 2016).

However, in the new millennium, the CoV outbreak occurred episodically by novel strains, for instance, SARS outbreak in 2002 by SARS-CoV strain and MERS outbreak in 2012 by MERS-CoV; both were examples of cross-species and spill-over infectious events. Due to the high genetic variation, a frequent occurrence in many species worldwide, and regular recombination events, CoV able to jump from the reservoir population to a novel population recurrently and thus are likely to appear in human-to-human (Cui et al., 2019). After the sequencing of the COVID-19 genome, it was found to be closely related to a β-CoV detected in bats belonging to the sarbecovirus subgenus and coronaviridae family (Zhu et al., 2020). This pleomorphic virus has a positive ssRNA surrounded by a spherical envelope forming a diameter of 60-100nm (Zhou M. et al., 2020). The rod-shaped S protein found on the viral surface is the main characteristic protein that is responsible for its antigenic properties (Zhu et al., 2020). The genome sequencing along with the other reports indicates that the COVID-19 genome is 75-80% similar to the SARS-CoV and 40% similar to the MERS-CoV genome (Lu R. et al., 2020). Although COVID-19, SARS-CoV, and MERS-CoV can grow in the same cells similarly in tissue culture COVID-19 is better grown in respiratory epithelial cells using human angiotensin-converting enzyme 2 (ACE2) like SARS-CoV (Zhou P. et al., 2020). It appears that the virus can transmit even after the manifestation of infection in the lower respiratory tract.

### Epidemiology

The emergence of infectious diseases since centuries threatens the existence of life and can be devastating unless appropriate measures and necessary initiatives are not taken to control outbreaks. However, monitoring and therapeutic management of microbes mainly viruses are very difficult due to their inherent nature of mutations and other genetic alterations (Bai et al., 2020; Srivastava et al., 2020). In late December 2019, the first case of CoV disease was detected in Wuhan city, Hubei Province, China, whereby local health facilities reported a cluster of patients with a primary diagnosis of pneumonia of unknown etiology (Bogoch et al., 2020; Lu H. et al., 2020). Initially, the Chinese authorities informed the cases of pneumonia to the WHO country office on December 31, 2019 (Lu R. et al., 2020). Later, the Chinese Center for Disease Control and Prevention isolated a novel CoV identified as the SARS-COV2 from the throat swab sample of an infected patient on January 7, 2020, which was genetically similar to SARS-CoV (Chen N. et al., 2020). Despite the recognition and extensive preventive measures, the infection rapidly spread in China within a short period and the outbreak extended in many countries of the world (Callaway et al., 2020).

Furthermore, the WHO confirmed that the COVID-19 was epidemiologically associated with the seafood and wet animal wholesale market of Wuhan (Sun et al., 2020). COVID-19 emerged in China where 82,447 people were infected with SARS-CoV2 and 3310 patients died with an overall mortality rate of 4% until March 30, 2020 (Baghizadeh Fini, 2020). According to the Chinese Center for Disease Control and Prevention, 81% of patients with COVID-19 had a mild case and 87% were 30–79 years old (Wu and McGoogan, 2020). The SARS-CoV2 infections are highly contagious and spreading exponentially across the world affecting about 11,560,845 people including 536,567 deaths in 215 countries on 1^st^ July 2020. As a global pandemic, COVID-19 has affected huge numbers of people across the African Region, Americas, Eastern Mediterranean Region, European Region, Southwest Asia, and Western Pacific Region of the world as shown in **Table 1**. As of March 30, 2020, there were 693,282 confirmed cases and 33106 deaths which further rise 15 times to 10357662 and 508,055 deaths worldwide until July 01, 2020. Further, the number of new cases increased drastically in the Americas and Southeast Asia; and the epicenter of the disease has subsequently shifted mainly to the USA, Brazil, and India.

**Table 1:** Newly reported and cumulative COVID-19 confirmed cases and deaths, by WHO Region (as of 06 September 2020) (WHO, 2020).

Regions	New cases in last 7 days (%)	Cumulative cases (%)	New deaths in last 7 days (%)	Cumulative deaths (%)	Mortality Rate
Americas	862,478 (46%)	14,001,390 (52%)	22 325 (59%)	484,079 (10%)	3,46
Southeast Asia	616,795 (33%)	489,943 (18%)	8,124 (22%)	83,400 (10%)	1.78
Europe	247,125 (13%)	4,475,267 (17%)	3,015 (8%)	222,279 (25%)	4.97
Eastern Mediterranean	92,699 (5%)	1,996,246 (8%)	2,244 (6%)	52,710 (6%)	2.64
Africa	38,639 (2%)	1,083,152 (4%)	1,207 (3%)	22,929 (3%)	2.12
Western Pacific	28,907 (2%)	516,478 (2%)	644 (2%)	11,206 (1%)	2.17
Others	–	741 (<1%)	–	13 (<1%)	1.75
**Globally**	**1,886,643 (100%)**	**26,763,217 (100%)**	**37,559 (100%)**	**876,616 (100%)**	**3.28**

As of September 06, 2020, in the United States of America, a total confirmed cases were 6,144,138 and 186,663 deaths, with 288,617 new cases and 5,974 deaths were registered in the last 7 days (WHO, 2020). Brazil recorded 4,092,832 positive cases, out of which 125,521 death cases were registered and the numbers peaked up to 288,029 confirmed cases and 6,017 deaths in the last week. In India, 4,113,811 confirmed cases, and 70,626 deaths were reported with 571,078 new cases and 7,128 deaths registered in the last 7 days (cluster of cases). In Indonesia, a total of 190,665 people were affected and 7,940 died due to this epidemic. In Bangladesh, 323,565 confirmed cases, and 4,447 deaths were reported with 14,640 new cases and 241 deaths in the last 7 days (WHO, 2020).

#### Epidemiological Characteristics of COVID-19

In late December 2019, an outbreak of SARS-CoV2 emerged in Wuhan, China and results in a cluster of COVID-19. As of April 2020, several studies were performed and detailed information regarding clinical characteristics obtained, which are important to understand the disease. Liang et al. (Tobaiqy et al., 2020) carried out a study between December 30, 2019, and January 31, 2020 and showed that a total of 457 patients were confirmed with COVID-19 of which male accounted for 267 (58%) and 98 (21%) patients had exposure to Huanan seafood wholesale market and record of contact with the infected person. Patient's signs and symptoms included fever (89%), cough (63%), and myalgia or fatigue (51%) with chief complaints of ARDS (60%). The most common comorbidities are cardiovascular and cerebrovascular diseases and hypertension (Słomka et al., 2020). In another report from China, these findings were corroborated. Guan et al. (2020) reported that the median age was 55 years and 48.9% were male and only 8 (3.6%) patients had a history of exposure to the Huanan Seafood Market. The most common symptoms in severe patients were high fever, anorexia, and dyspnea. As of February 15, 2020, 19% of patients had been discharged and 5.4% of patients died. Furthermore, 80% of severe cases received intensive care unit (ICU), and the mortality rate in ICU was 20.5% (Guan et al., 2020). Similar results were obtained in another study among COVID-19 patients admitted with major complications of ARDS (48.6%) in the Central Hospital of Wuhan, China from January 2 to February 1, 2020.

Liu Y. et al. (2020) showed among patients, the mean age was 55 years, and 59 (54%) patients were male. With a median 15 days (range, 4 to 30 days) follow-up period, 31 patients (28.4%) died, while 78 (71.6%) survived and discharged. ARDS patients were elder (mean age, 61 years) and more likely to have comorbidities including diabetes (20.8%), cerebrovascular disease (11.3%), and chronic kidney disease (15.1%) (Liu Y. et al., 2020). The findings of Zhou F. et al., (2020) showed that out of 191 patients (135 from Jinyintan Hospital and 56 from Wuhan Pulmonary Hospital), 137 (71%) were discharged and 54 (28%) died in the hospital. 91 (48%) patients had comorbidity, with hypertension being the most common (30%), followed by diabetes (19%) and coronary heart disease (8%). The median duration of viral shedding was 20 days in survivors, and the longest observed duration of viral shedding in survivors was 37 days. Mo et al. (2020) showed in addition to older age, male sex, more underlying comorbidities, the COVID-19 patients reported high levels of neutrophil, aspartate aminotransferase (AST), lactate dehydrogenase (LDH) and C-reactive protein, lower levels of platelets and albumin, and higher incidence of bilateral pneumonia and pleural effusion on admission. The epidemiology of COVID-19 provides a better understanding of the pattern of disease growth and spread. The virus is of zoonotic origin and has the potential of causing infection from human to human transmission by means of contact, large droplets and can also be transmitted through aerosols and cause infection (Menachery et al., 2018; Perlman, 2020). Safety measures such as public awareness, social distancing and maintenance of hygiene are important ways to protect from SARS-CoV2 infection. However, Government of the People Republic of China successfully controlled COVID-19 by maintaining social distancing through long and strict lockdown and curfew nationwide. The European countries and the USA have become the epicenter of the disease as because not taking issues seriously. High and densely populated countries like India and Bangladesh should pay special attention and implement strict policies to control COVID-19 otherwise the outbreak of the disease can lead to a devastating situation. Since no treatment is available, precautions like maintenance of physical distancing and proper hygiene would help further spread of COVID-19.

## Symptoms of COVID-19

**Figure 1:**
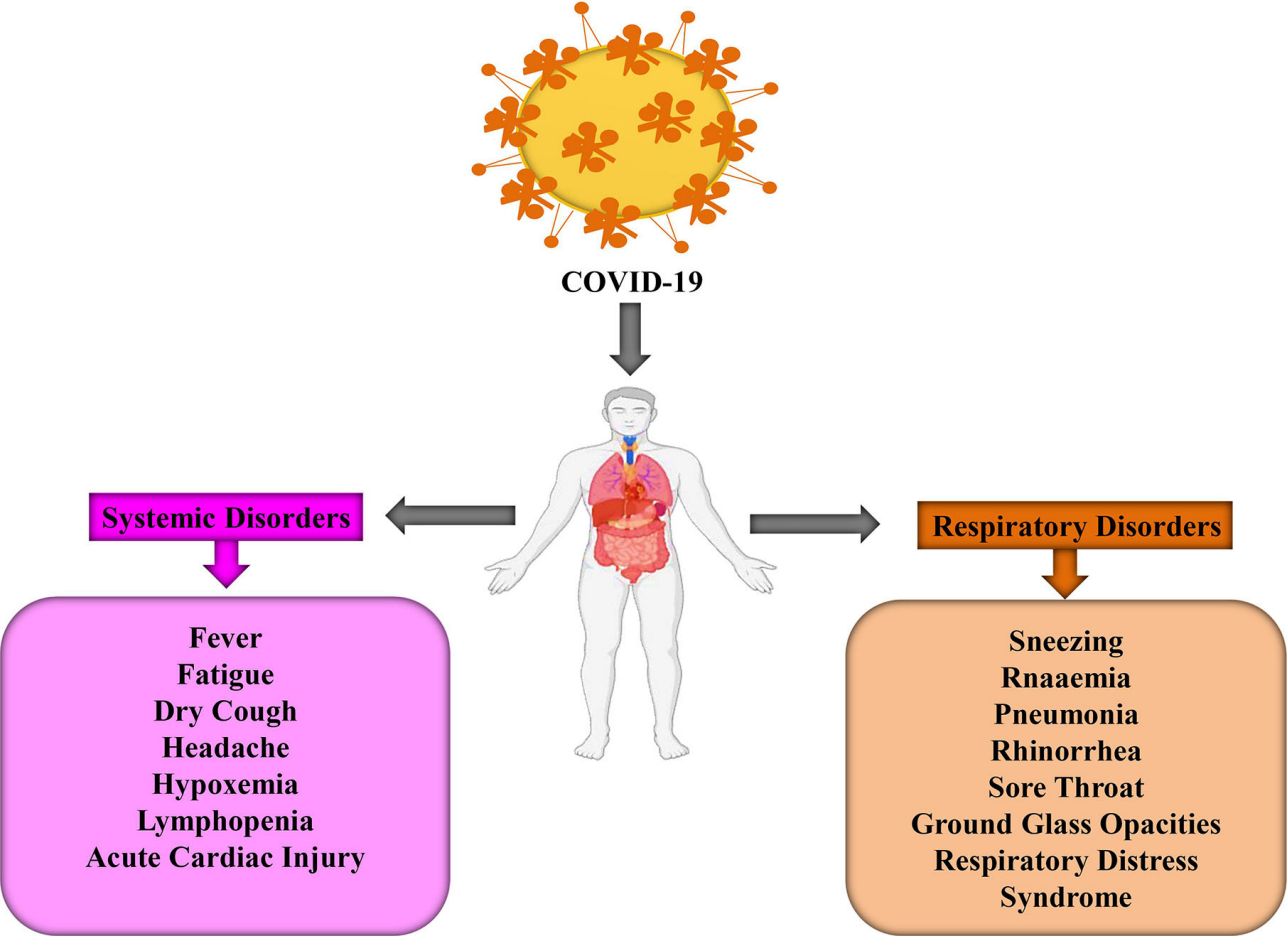
The systemic and respiratory disorders in COVID 19 infected patients.

COVID-19 infected people may arise their symptoms within 2 to 14 days but sometimes, in some cases, this disease prevails after 27 days. However, a group of Chinese researchers revealed that the average incubation time is approximately 5.2 days (Li Q. et al., 2020). During this period, there is no significant change in peripheral blood leukocytes (PBL) and lymphocytes. Generally, the viruses spread out in the lungs, heart, GIT, and through the bloodstream. Primary lesions become noticeably worse around 7–14 days and PBL reduces significantly, including both T and B-lymphocytes (Li T. et al., 2020). The time frame for COVID-19 patients’ symptoms to death has been observed 6 to 41 days where the median period is 14 days (Wang W. et al., 2020). Although this period depends on two crucial factors, namely, the patient’s age and immune function. It is important to note that the number of cases is higher in above 70 years people in comparison with the person below 70 years (Wang W. et al., 2020). Several symptoms, including fever, dry cough, fatigue, muscle pain, sneezing, headache, hemoptysis, dyspnea, sputum production, lymphopenia, sore throat, and respiratory problems are developed after infecting COVID-19 (**Figure 1**) (Graham Carlos et al., 2020; Huang C. et al., 2020; Ren et al., 2020). To be more specific, these symptoms are divided into three categories: most common symptoms (i.e., fever, dry cough, and tiredness), less common symptoms (i.e., aches and pains, conjunctivitis, sore throat, diarrhea, headache, loss of taste or smell, a rash on the skin, and discoloration of fingers and toes), and serious symptoms (i.e., difficulty during breathing or shortness of breath, chest pain or pressure, and loss of speech or movement). The risk of COVID-19 is higher in children, older people, and some patients who are suffering from diabetes, cancer, heart diseases, and lung diseases (Ali and Alharbi, 2020). COVID-19 patients experienced gastrointestinal (GI) symptoms including diarrhea, whereas a low rate of either SARS-CoV or MERS-CoV patients developed similar GI symptoms. As a result, it is urgent to analyze urine and fecal symptoms to exclude an effective transmission route, through doctors, nurses, and patients (Lee et al., 2003). Some patients are reported with respiratory distress syndrome while critical patients require intensive care because of respiratory failure, septic shock, and organ failure (Lee et al., 2003; Chen N. et al., 2020; Huang C. et al., 2020).

Additionally, the chances of COVID-19 are greater if there is a loss of breath, cough, and some individuals who contacted a COVID-19 infected person or traveled the COVID-19 affected zone. Under this circumstance, the clinical test should be applied among those people and infected zones must be locked down. However, some COVID-19 infected patients recover easily because of their high immunity power and bring back normal conditions while others may need extra time due to health conditions or age limitations (i.e. early age or late age) (Ali and Alharbi, 2020).

## Transmission of COVID-19

**Figure 2:**
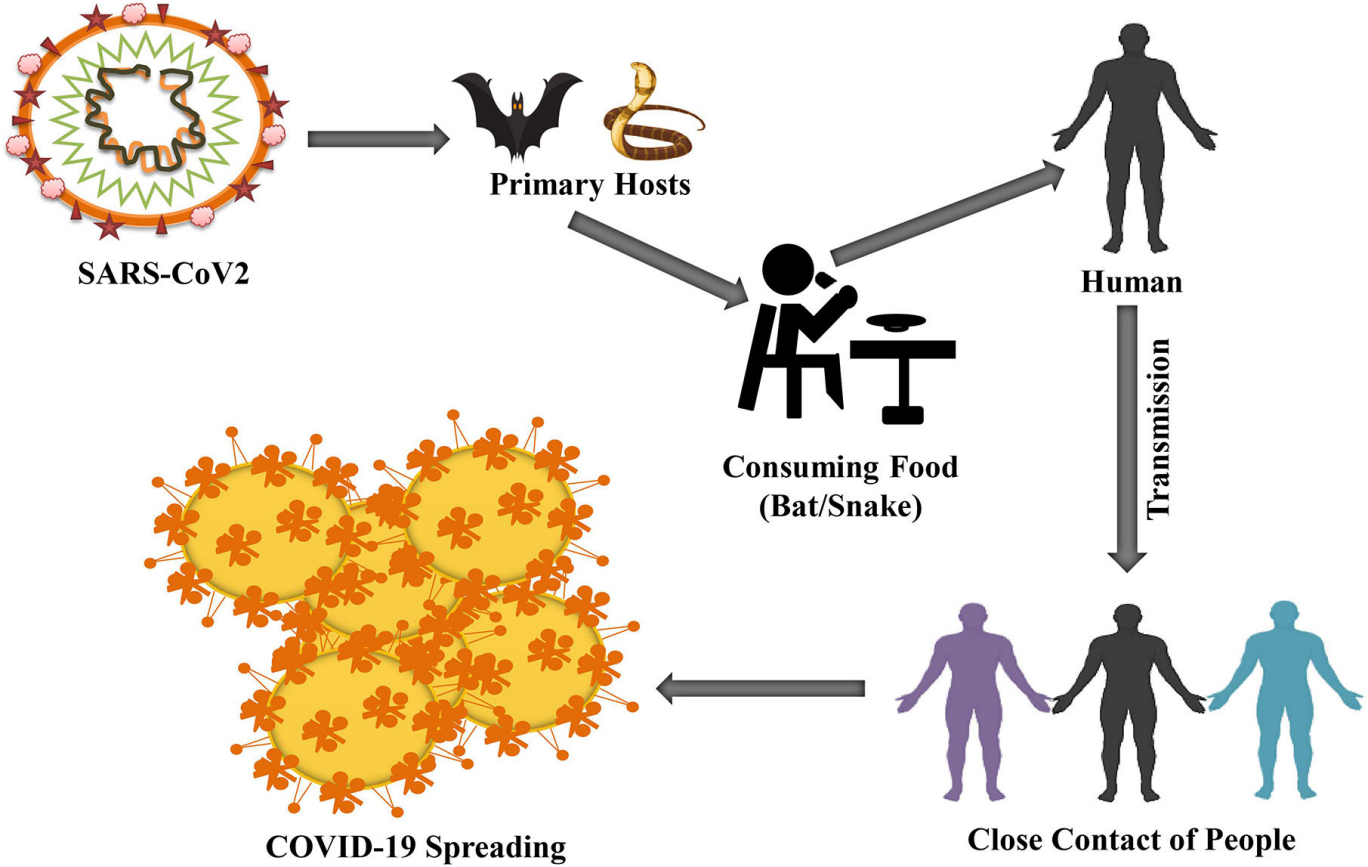
Humans are primarily infected by consuming suspected reservoirs (bats or snakes). The possibility of COVID-19 is increased because of close contacts, such as handshaking from human to human.

A variety of measures had been taken to find out a specific reservoir host or carriers from Hubei province, China where COVID infected people were first found. The initial investigation confirmed two snake species as a possible reservoir of novel COVID-19. However, until now, there is no exact evidence of COV reservoirs other than birds or mammals (**Figure 2**) (Bassetti et al., 2020; Ji et al., 2020). The genomic sequence of COVID-19 exhibited 88% identity with bird-derived SARS-like COV (Lu R. et al., 2020; Wan et al., 2020) ensuring a link between humans and COVID-19.

Suspended respiratory droplet generated from coughing and sneezing is one of the recognized media of transmission of COVID-19 from human-to-human. Intra-human spreading is recorded in different countries around the world (Li Q. et al., 2020). The pattern of transmission known through the established cases generated mass panic and fear among people across the globe. Moreover, the possibility of transmission through the oral-fecal route is still not turned down. The current practice to prevent the disease is to supportive and palliative care while isolating the affected and keeping the suspected corona patients to whom he/she contacted in quarantine. There is no way to keep away from the virus if someone does not stay away from the patient infected with COVID-19. Respiratory infections, including COVID-19 infection, are transmitted through a variety of sized particle droplets. Besides, >5–10 diameter of droplet particles is considered as respiratory droplets, while <5–10 diameter of droplet particles is considered as droplet nuclei (Chan et al., 2020b). Current evidence assures that respiratory droplet and contact routes are significantly associated with COVID-19 transmission (Liu J. et al., 2020). Noteworthy, droplet transmission spreads if a person contacts within 1 m distance with someone who exposes respiratory symptoms such as sneezing and coughing (Li Q. et al., 2020). Thus, infective respiratory droplets elevate the risk of spreading COVID-19 into mucosa (mouth and nose) and conjunctiva (eyes). This virus can persist in cough or sneezing droplets for 2 h to a few days (Ali and Alharbi, 2020). The transmission of COVID-19 may also happen by fomites in the intermediate environment around the COVID-19 infected patients (Ong et al., 2020). Therefore, it has been demonstrated that the transmission of COVID-19 can occur either direct contact with an infected person or indirect contact with used medical accessories (i.e., stethoscope or thermometer) or medical wastes, including COVID-19 infected gowns, gloves, or facial masks.

In this pandemic situation, frontline healthcare workers (FHWs) are highly recommended by WHO to use PPE, including N95 respirator mask, gloves, gown, face shield, and goggles (Tabary et al., 2020). Different types of polymers such as latex, polyurethane, nitrile rubber, neoprene, and polyvinyl chloride are used to make medical gloves. Furthermore, nitrile and latex gloves have been considered as a better choice during the COVID-19 pandemic because these gloves show better durability (Kwon et al., 2020). Besides, a study performed in Singapore’s 41 FHWs in close contact, for 10+ min, <2 meters away, with critically infected COVID-19 patients exhibited no transmission because FHWs wore N95 respirator mask, gloves, gown, face shield, and goggles properly (Ng et al., 2020). This study confirms that adequate protection contributes to reducing the risk of COVID-19 transmission. However, some people, including cleaner, trash collectors, and day laborers, are at higher risk of COVID-19 due to their regular contact with medical wastes (Saadat et al., 2020). It can be predicted that those workers might be infected during carrying COVID infected medical wastes because this virus can survive 2 h to few days in sneezing droplets on the surface. Furthermore, some medical wastes, including N95 respirator mask, hand sanitizer bottles, and their rapping solid tissue papers, are one of the most risk factors for transmitting COVID-19 that may be a threat not only for humans but also for natural habitat in land and ocean (Hellewell et al., 2020). For instance, Ocean Asia NGO surveyed (Hellewell et al., 2020) in Hong Kong in late January 2020, and they observed that 7 million people wear one or a couple of masks, gloves, and hand sanitizers every day. As a result, the amount of trash is increasing dramatically, which leads to polluting the environment (Saadat et al., 2020).

Another study demonstrated that sometimes ocean fishes mistakenly eat medical wastes as food and they fall in death (Hellewell et al., 2020). Li Y. et al. (2020) reveal that COVID-19 is predominantly transmitted by symptomatic patients to others who contact with infected patients or close contact with respiratory droplets. Furthermore, a family of three members who traveled from COVID identified place Wuhan to Guangzhou during the epidemic of COVID-19 (Pan et al., 2020) and after clinical investigation, the father showed an abnormal chest CT image, a reduced lymphocyte count, and RT-PCR positive. Interestingly, mother and son were asymptomatic along with normal chest image and normal lymphocyte count, but RT-PCR was positive. Similarly, a 10-year-old child traveled from Shenzhen to Wuhan and after returning Shenzhen, the child was asymptomatic, but a few days later, the full family was affected in COVID-19 (Fuk-Woo Chan et al., 2020). In fact, in places where the epidemic blowup, social distancing among the healthy people and special separation for the infected people or isolation measures must be taken. Some daily hygiene practices such as washing hands regularly with soap water, using the face mask, a social distancing that is avoiding gatherings, staying away from unknown persons, poultry, or cattle farms, and maintaining a good food practice such as eating fully cooked egg or meat are highly recommended by WHO (2020).

## Multiorgan Damage by COVID-19

Primarily, COVID-19 is known as a respiratory illness whereby the lungs and lower respiratory tracts are mainly affected, and infected patients are clinically manifested by dry cough, fever, shortness of breath, and/or dyspnea (Subbarao and Mahanty, 2020). The novel coronavirus can also attack several organ systems and can adversely affect the heart, kidneys, liver, nervous system, blood vessels, and skin. The underlying condition in many patients with extrapulmonary disease is thought to be cytokine storm in which the excessive release of cytokine can cause cellular, tissue, and ultimately organ damage (Sungnak et al., 2020). COVID-19 has been known to be implicated in multiorgan system damages. The lungs, heart, nerves, brain, vessels, kidneys, and skin affected by the SARS-CoV-2 pathogens are discussed herein.

### Brain and Nervous System

According to previous studies, CoV can easily penetrate the brain via the nerve cells (Baig, 2020; Zubair et al., 2020). Researchers found that the nerve cells serve as a gateway for the virus into the central nervous system (CNS) and damage the respiratory center resulting in irregular breathing (Ahmed et al., 2020). Additionally, SARS-CoV-2 affects the nervous system, and patient with severe COVID-19 is also associated with confusion, lethargy, disorientation, decreased consciousness, and acute necrotizing hemorrhagic encephalopathy (Mao et al., 2020). These findings are consistent with cytokine storm syndrome. Of note, around 80% of COVID-19 patients lose their sense of taste and smell. At the early stage of infection, ageusia or anosmia occurs. Depending on these symptoms, a person is suspected as a COVID patient and distinguished for further investigations. This observation reveals that the nervous system of COVID-19 patients is affected (Koralnik and Tyler, 2020). However, these symptoms can be raised by adenovirus at an advanced stage but not an early stage. In addition, about 50% of hospitalized COVID-19 patients have headaches, ageusia, or anosmia (Asadi-Pooya and Simani, 2020). In Italy, about a fifth of cases exhibited the same symptoms, while around 90% of patients had ageusia or anosmia in Germany (Zubair et al., 2020). Furthermore, patients who suffer from COVID-19 showed neurological disorders such as seizure, confusion, cognitive impairment, memory loss, dizziness, and/or coma. A case study performed in Japan reveals that COVID-19 patients developed encephalitis and meningitis, resulting in tissue damage into the brain (Moriguchi et al., 2020). Similarly, a study has performed in Wuhan, China assures that about 45% of COVID-19 patients who are critically ill show neurological deficits. Another study in France stated that 84% of ICU admitted COVID-19 patients had positive abnormalities in the brain, and 15% of patients faced cognitive impairment and difficulty in making decisions (Fotuhi et al., 2020).

### Heart

**Figure 3:**
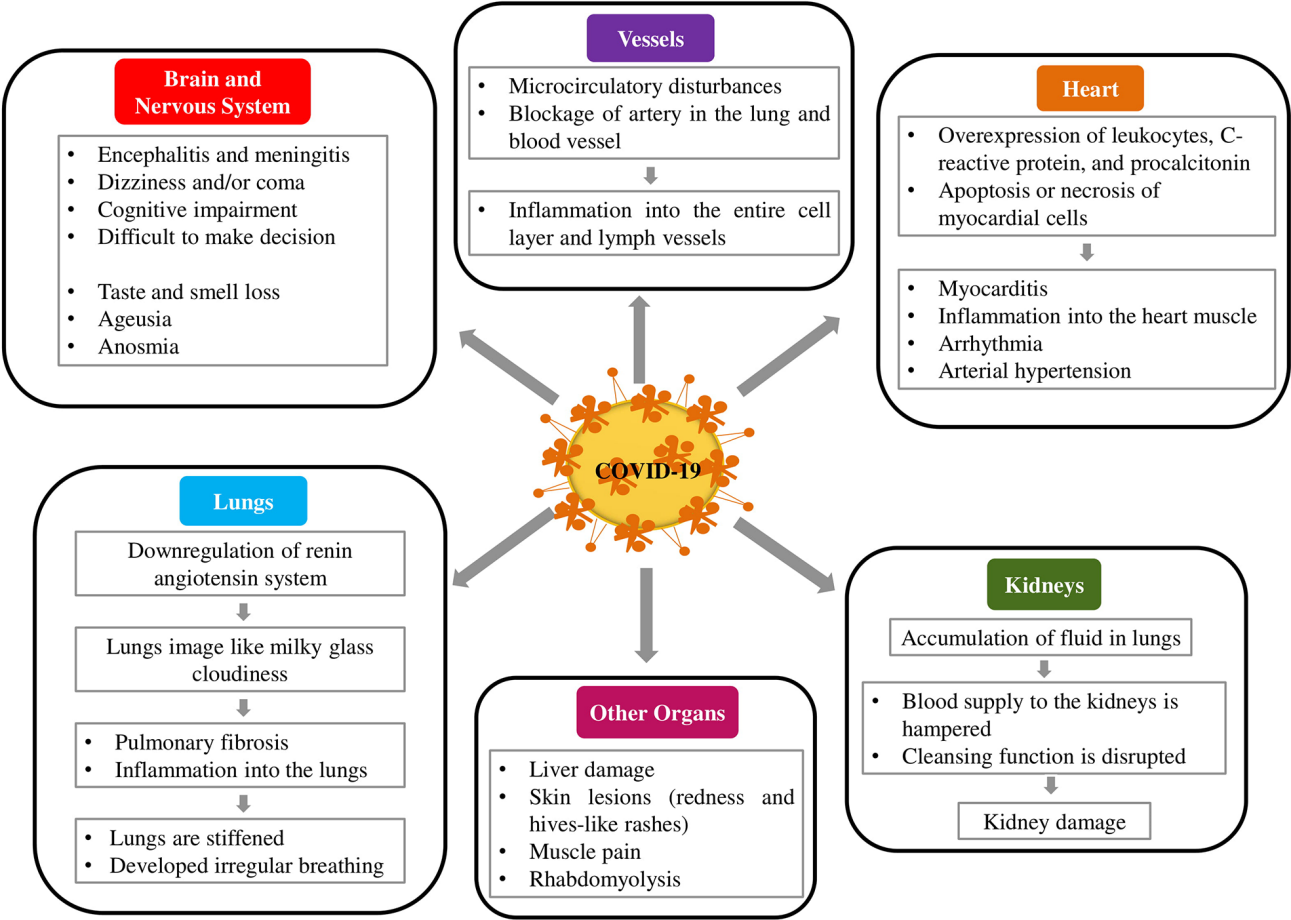
COVID-19 imparts adverse implications on human organs, including lungs, brain, heart, and kidney.

It has been well established, viral infection contributes to cardiac injury (Esfandiarei and McManus, 2008; Corsten et al., 2012). SARS-CoV-2 can easily attack the heart of those patients who are already suffered from cardiovascular disease (Wu L. et al., 2020). Furthermore, high blood pressure is one of the most common risk factors of COVID-19 infected patients (Hafiane, 2020). Recent studies has showed that patients who are critically ill with COVID-19 elevate severe inflammation into the heart resulting in damage heart muscle (Guo J. et al., 2020; Wang D. et al., 2020). This inflammation also causes rhythm disturbances and muscle damage. In addition, numerous studies from various countries, including China, Italy, and the USA, indicate that COVID-19 attacks the heart and causes myocarditis and inflammation into the heart muscle (Madjid et al., 2020; Shi et al., 2020). Inflammatory markers, such as leukocytes, C-reactive protein, and procalcitonin are significantly enhanced among COVID-19 patients who are in cardiac injury. These elevated levels of cytokines and inflammatory markers are associated with either apoptosis or necrosis of myocardial cells (Shi et al., 2020). A study published in JAMA Cardiology assured heart damage around 20% of 416 patients COVID-19 in China, and another study revealed that 44% of patients out of 36 hospitalized to the ICU faced arrhythmias (Shi et al., 2020). Furthermore, among 184 COVID-19 infected patients in Dutch ICU, 38% of patients’ blood clotted abnormally, according to a study published in Thrombosis Research (Klok et al., 2020). Clots from the arteries can lead to lodge in the brain that causes a stroke. Blood clots block vital arteries observed in COVID-19 patients. Similarly, according to a recent report, 25 and 58.3% of patients are critically suffered from heart disease (**Figure 3**) and hypertension, respectively (Wang D. et al., 2020).

### Lungs

The most frequent clinical manifestation of COVID-19 disease is pneumonia, which is characterized by cough, fever, and bilateral infiltrations (Zhou S. et al., 2020). Subsequently, shortness of breathing, reduced physical work, and even daily activities become difficult. Primarily, COVID-19 attacks the lungs and then reduces lung functions. A group of Chinese researchers reported in their study that the image of COVID-19 patients’ lungs was like milky glass cloudiness (Ahn et al., 2020). Some patients have recovered from COVID-19 but not reduced the cloudiness from the lungs. This result suggests that the organ is damaged permanently. Furthermore, some patients faced pulmonary fibrosis during the progression of COVID-19, which results in inflammation into the lungs (Xia et al., 2020). As a result, the oxygen is not reached properly to the blood vessels, lungs are stiffened and developed irregular breathing, and physical performances are also hampered (Lai et al., 2020).

### Vessels

Abnormal blood coagulation in patients with COVID-19 has been reported. Intravascular coagulation is very common and occurs in 71.4% of patients who died in COVID-19 (Tang et al., 2020b). In some COVID-19 patients, the endothelium of blood vessels and lymph from various organs demonstrated inflammation as evidenced by the autopsy of deceased patients. It was found that generalized inflammation in the endothelium solely caused by SARS-CoV-2 via ACE2 receptors (Zaim et al., 2020). Some pathologists from the University Hospital of Zurich analyzed the autopsy of deceased COVID-19 patients, and they found that the entire cell layer and lymph vessels of several organs became inflamed (Varga et al., 2020). The researchers suggested that COVID-19 played a crucial role in progressing generalized inflammation via the ACE2 receptor in the endothelium (Behl et al., 2020). This could enhance microcirculatory disturbances that directly damage the function of the heart and also cause the blockage of an artery in the lung and blood vessels in the brain and the intestinal tract. Therefore, multiorgan failure happens, which results in severe illness to death.

### Kidneys

The main function of the kidney is to excrete various waste products from the body. However, a recent study reveals that abrupt loss of kidney function develops shortly with COVID-19 infection (Naicker et al., 2020). Although COVID-19 is thought to occur due to acute tubular necrosis, the exact mechanism of kidney damage is unknown. However, research indicated that ACE2 receptor expressed in kidney cells (Xu X. W. et al., 2020) and SARS-CoV-2 might be responsible for direct renal cellular damage, which was further supported by the detection of SARS-CoV-2 in urine from an infected patient (Naicker et al., 2020). Acute kidney failure often occurs in COVID-19 patients with pneumonia. Data showed that about 30% of patients with COVID-19 required dialysis or continuous renal replacement therapy (Gedney, 2020). COVID-19 patients with pneumonia accumulate lots of fluid in the lungs. As a result, the blood supply to the kidneys is hampered, and kidneys cannot perform their cleansing function properly. Diao et al. (2020) revealed that 27% of 85 hospitalized COVID-19 patients had kidney failure in Wuhan. Another study demonstrated that 59% of 200 patients who were ill with COVID-19 in Hubei and Sichuan provinces had underlying kidney damage (Volunteers et al., 2020). Small infarctions were observed in kidney tissue who suffered from COVID-19. COVID-19 along with acute kidney injury patients were died more and more rather than acute kidney injury patients.

### Other Organs

Approximately 50% of all COVID-19 patients reported GI symptoms such as diarrhea, nausea, vomiting, and abdominal pain (Wong et al., 2020). The incidence of GI symptoms, alongside the detection of SARS-CoV-2 RNA in stool samples of infected patients (Holshue et al., 2020), suggest that ACE2 receptors highly expressed in the GI tract are another target for SARS-CoV-2 infection. Mild and transient liver injury, as well as severe liver damage, can occur in COVID-19 infected patients. Recent results indicated that around 15–55% of COVID-19 patients had elevated levels of alanine aminotransferase, aspartate aminotransferase, bilirubin, and gamma-glutamyl transferase (Wong et al., 2020; Zhang C. et al., 2020). Furthermore, they reported that the extent of liver damage is proportional to the severity of the SARS-Cov-2 attack (Wong et al., 2020). COVID-19 patients showed significant skin lesions caused by SARS-CoV-2. On toes, small skin lesions have also been observed particularly in children and young people and the lesions resembled as redness, marks, and hives-like rashes have also been observed. The muscle pain is a very common symptom of COVID-19 (Kucuk et al., 2020). A single case of rhabdomyolysis has been reported as a consequence of COVID-19 (Jin and Tong, 2020).

### Inflammatory Markers

If the immune system fails to beat back this novel virus during the initial stage, firstly, it attacks the lungs and later, spreads out the other parts of the body and damages other organs, including the brain, gut, heart, intestine, and kidney (Zhang J. et al., 2020). Chen L. et al. (2020) revealed that the levels of inflammatory cytokines increased in COVID-19 infected patients, and the real morbidity and mortality of this disease was also changed due to elevated levels of inflammatory cytokines. Furthermore, Huang Y. et al. (2020) demonstrated that ICU admitted COVID-19 patients showed a higher level of granulocyte-colony stimulating factor, monocyte chemoattractant protein-1, chemokine ligand 3, IgG-induced protein 10, tumor necrosis factor α (TNF- α), and plasma cytokines, including interleukin (IL)-2, IL-6, IL-7, and IL-10. Similarly, the levels of pro-inflammatory cytokines, including TNF- α, IL-1β, IL-6, IL-8, GM-CSF, and G-CSF and chemokines, including IP10, MCP1, and MIP1α are increased in COVID-19 infected patients (Schett et al., 2020). Messina et al., (2020) revealed that elevated levels of IL-6 and TNF-α were found in COVID-19 infected patients. IL-6, an endocrine cytokine, is associated with exacerbating viral disease and overexpression of this pro-inflammatory cytokine promotes virus survival time during viral infection (Velazquez-Salinas et al., 2019; Monda et al., 2020). In addition, IL-6 also activates the JAK/STAT3 signaling pathway that results in apoptosis and cell death. On the other hand, TNF-α significantly promotes the expression of c-JNK and NF-κB, and the overexpression of TNF-α is associated with progressing viral diseases including COVID-19 (Guo Y. R. et al., 2020).

Autopsy should be considered as the gold standard to elucidate the exact cause of death, thus provide information on the pathogenesis of the COVID-19 infection and to take necessary measures to control the infection. Several retrospective clinical studies were carried out to define the specific cause of death in patients with COVID-19; however, the available data only suggesting the gross involvement of the organs (Wu C. et al., 2020; Yang et al., 2020). On the contrary, the autopsy provided clinically-relevant insight that is the progression of the unknown disease condition as reported by Ng et al. (2016). In fact, the physicians and researchers should have a clear idea of route cause that affecting the vessels of different organs such as the lung, heart, kidney, liver, intestine, CNS, and even the skin. To understand the precise mechanism underlying death due to COVID-19 infections, the importance of autopsy as a diagnostic tool cannot be overlooked (Salerno et al., 2020).

Despite the improvement in modern diagnostic techniques and real-time intensive monitoring, miss diagnosis reports among ICU deceased has not essentially changed over the last 30 years (Roosen et al., 2000), whereas autopsies reduced ante-mortem diagnostic errors in about 30% of death cases (Combes et al., 2004; Kotovicz et al., 2008). At this critical stage, the practice of medical science encouraging autopsy as a tool to learn from inevitable deaths that would help the clinicians to choose an effective therapeutics to reduce mortality. However, the management of the COVID-19 survivor is the next challenge for the scientific community. It is a matter of great concern that a COVID-19 who luckily survived after viral infection with a period of hospitalization and extensive drug treatment require further regular medical follow-up for the organs involved and/or damaged as the late consequences of the infection. Therefore, our knowledge regarding the current management of the infection should be updated and proper guidelines for the medical care of COVID-19 survivors should be established.

## Therapeutic Interventions for COVID-19

### Anti-Viral Treatment

#### Lopinavir

Lopinavir (LNV) along with ritonavir (RTV) is extensively used as an enriched protease inhibitor in treating human immunodeficiency virus (HIV) infections (Tsang and Zhong, 2003; Kumar et al., 2020). By showing antiviral activity, LNV mitigates MERS-CoV infected disease progression in the marmoset model. However, the antiviral action of LNV is unclear against MERS-CoV in *in vitro* model (Arabi et al., 2018). LNV is generally coupled with RTV to improve the half-life of LNV through cytochrome P450 inhibition (Sheahan et al., 2020). Chu and others (Chu et al., 2004) demonstrated that the use of RVN combined with LNV/RTV exhibited a better inhibition on the progression of SARS. Furthermore, Kim et al. (2016) revealed that triple combination therapy including LNV/RTV, INF-α 2a, and RTV had remarkable outcomes during the outbreak of MERS-CoV in South Korea. So, Kim’s triple therapy can be considered as a therapeutic candidate at the early stage of COVID-19. It is unfortunate to mention that some patients were died at the late stage of COVID-19, although they received LNV/RTV. Moreover, some studies suggest that the LNV/RTV concentration is necessary to suppress pulmonary SARS-CoV2 replication might be higher than the serum level (Cao et al., 2020; Sheahan et al., 2020). In 2020, a randomized, controlled open-label trial was performed to identify the efficacy of LNV/RTV at a dose of 200/50 mg twice a day among hospitalized patients who were critically infected in SARS-CoV2 in china (Cao et al., 2020). Additionally, the administration of LPV/RTV combined with ribavirin is effective against patients with SARS-CoV as well as in tissue culture (Chu et al., 2004).

#### Remdesivir

Remdesivir (RDV) is an antiviral drug belonging to nucleoside analog GS-5734, which integrates into nascent RNA chains and contributes to causing their premature termination. RDV has been widely reported to suppress the pathogenesis of human as well as zoonotic CoV *in vitro* and to prevent SARS-CoV *in vivo* (Agostini et al., 2018). Several clinical trials are going on to evaluate the effectiveness as well as the safety of RDV in COVID-19 patients. Recently, the US FDA has approved RDV as an emergency use for COVID-19 patients. In a randomized, controlled trial involving 1063 patients, RDV treated patients need to shorter time (11 vs. 15 days; p<0.001) to recover from COVID-19 and showed lower mortality rate (8% vs. 12%; p=0.059) compared to the placebo group (NIH, 2020). However, Wang Y. et al. (2020) reveal that statistically, RDV does not show significant clinical benefits in adult COVID-19 hospitalized patients. Recent evidence has revealed that the antiviral activity of RDV and IFN-β is superior to LNV/RTV-IFN-β for preventing MERS-CoV *in vitro* as well as *in vivo* (Sheahan et al., 2020). Furthermore, in mice model, RDV could boost pulmonary function, attenuate pulmonary viral loads, and extreme lung pathology which was not possible for LNV/RTV-IFN-β (Sheahan et al., 2020). In the USA, recently RDV has administered in COVID-9 infected patients when the clinical condition of patients is getting worsen (Holshue et al., 2020). Consequently, the use of RDV with IFN-β can be a better alternative to the combination of triples LNV/RTV-IFN-β for the treatment of COVID-19. A randomized and controlled trial should be required to assess the safety and efficacy of RDV along with IFN-β in COVID-19 infected patients.

#### Ribavirin

Ribavirin (RVN) is referred to as an antiviral agent, which is predominantly used for the treatment of hepatitis C (HP-C). In Hong Kong during the SARS outbreak, RVN was widely used in most cases with or without the concomitant use of steroids (Wenzel and Edmond, 2003). However, there has been substantial skepticism about the efficacy of RVN from overseas and local experts (Cyranoski, 2003). As RVN has been reported to have no significant action against SARS-CoV *in vitro*, it has also been found that the use of RVN is associated with substantial toxicity including hemolysis (76%) and a reduction in hemoglobin (49%) (Booth et al., 2003). Nevertheless, Morgenstern et al. (2005) indicated that RVN combined with IFN-β showed synergistic inhibition of SARS-CoV replication not only animal models but also human cell lines. In contrast, RVN combined with several antiviral drugs is still under clinical trials because these drugs show poor efficacy and an unpleasant adverse reaction. Although the poor in vitro efficacy and the unfavorable adverse reaction profile, however, the potential therapeutic use of ribavirin in combination with other antiviral therapies is still under investigation.

#### Arbidol

**Figure 4:**
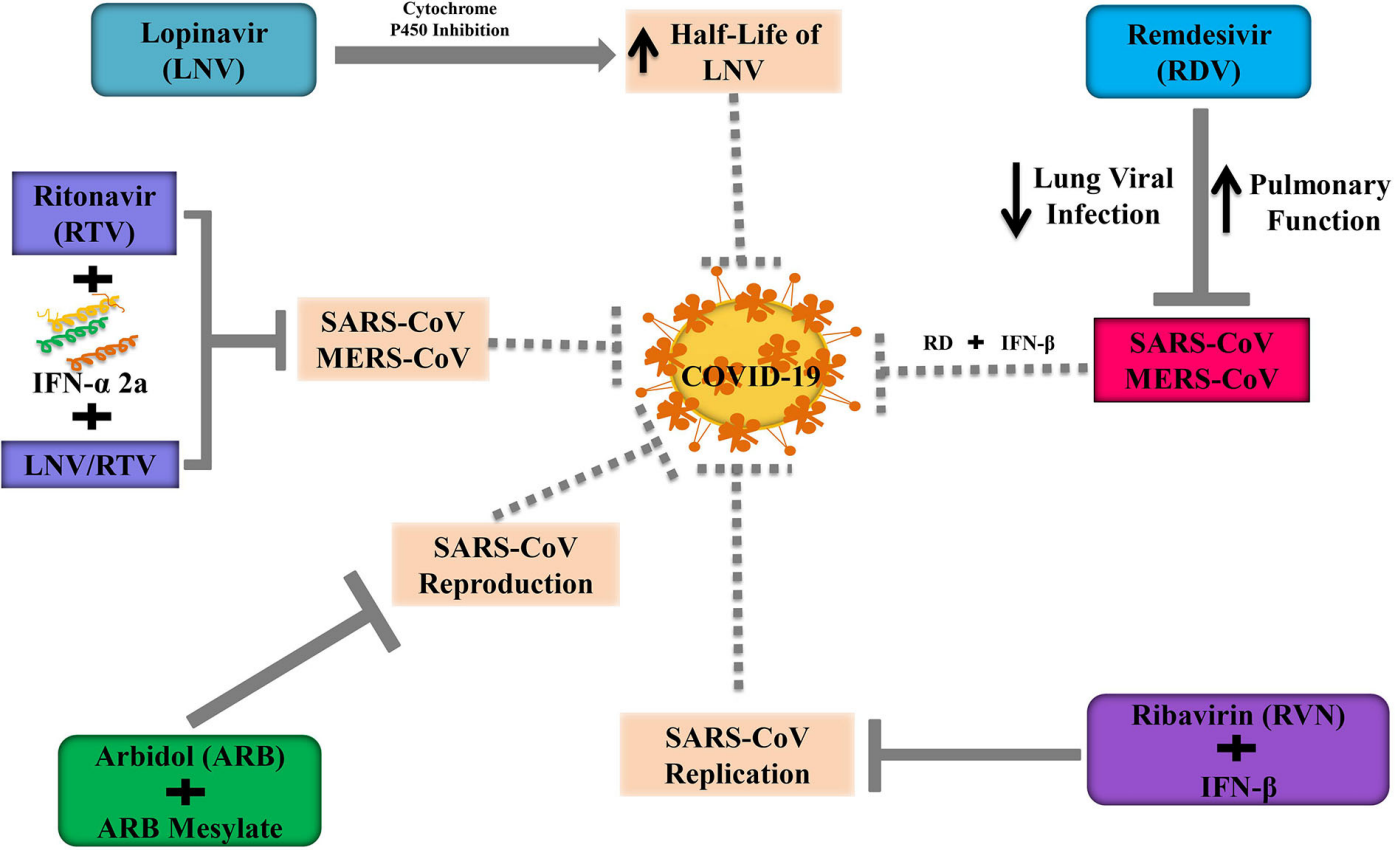
Anti-viral candidates inhibit the replication and reproduction of SARS-CoV and MERS-CoV.

Arbidol (ARB) is a small indole-derivative compound, which is firstly licensed in Russia and China for the prevention and treatment of influenza and other respiratory viral infections (Blaising et al., 2014). ARB can block viral infusions against the HP-C virus and influenza A and B (Boriskin et al., 2008). ARB has been found to suppress HP-C by blocking the entry gate and replication in *in vitro* model (Pécheur et al., 2016). Moreover, ARB and its derivatives such as arbidolmesylate exert antiviral effects against the SARS pathogen in cell cultures, and it is interesting to note that arbidolmesylate is five times more effective than ARB to alleviate SARS reproduction in *in vitro* model (**Figure 4**) (Khamitov et al., 2008).

#### Epigallocatechin Gallate

Epigallocatechin gallate (EGCG), a polyphenolic compound derived from green tea, plays distinct antiviral effects in *in vitro* and *in vivo* model (Ling et al., 2012). EGCG can significantly inhibit viral protein expression and host factors. A recent study revealed that antiviral activities of EGCG affect influenza virus infectivity in cell culture (Weber et al., 2003). EGCG has also been reported to agglutinate and inactive the virus through the deformation of phospholipids. Besides, intracellular compartments (endosomes and lysosomes) acidification are inhibited by EGCG resulting in suppression of influenza virus growth in *in vitro* model (Imanishi et al., 2002). EGCG also inhibits the replication of influenza A with respect to concentration and time-dependent manner (Ling et al., 2012). However, oxidative stress and elevated ROS generation are the hallmarks of viral infection. Interestingly, EGCG has highly free radical scavenging power (potent antioxidant), which regulates ROS production and oxidative stress (Jackson et al., 2010; Vlahos et al., 2011). Recent studies indicate that EGCG is considered an inhibitor of the HP-C virus and identified as an antihemeolytic agent. EGCG combined with Dactavira and/Dactavira plus may impede the mechanism of viral entry and play a pivotal role in preventing relapse as enhancing efficacy in comparison with the standard of care (Shiha et al., 2019). Khan et al. (2020) revealed that EGCG, a lead compound, which can easily bind with the docked proteins of SARS-CoV2 and EGCG experienced to make molecular interrelation with binding energies -4.90, -6.99, -7.26, -7.57, -8.38, -8.66, and -9.30 kcal/mole for 6lvn, 6lu7, 6vsb, 6lxt, 6vww, 6vw1, and 6y2eproteins of SARS-CoV2, respectively. So, these results strongly suggest that EGCG treatment should be explored as a potential therapeutic target to alleviate COVID-19 after considering the antiviral activities of EGCG.

#### Favipiravir

Favipiravir (FPV) was first discovered, at Toyama Chemical Co., Ltd research laboratory, by phenotypic screening for antiviral activity against influenza virus (Furuta et al., 2017). FPV is considered as an antiviral agent, which inhibits RNA viruses selectively and potently. FPV undergoes phosphorylation to make an active form, FPV ribofuranosyl-5B-triphosphate, which is confessed as a substrate by RNA-dependent RNA polymerase (RdRp) and suppresses the activity of RNA polymerase. FPV is crucially effective against several types and subtypes of influenza viruses, for example, strains resistant to anti-influenza drugs (Furuta et al., 2013; Jin et al., 2015). FPV ribofuranosyl-5B-triphosphate exerts no effects on either DNA-dependent RNA (DdR) or DNA polymerases. These features demonstrate that FPV favors RNA virus rather than DNA virus and mammalian cells. Moreover, FPV exhibits antiviral actions against a wide range of RNA viruses, including bunyaviruses, arenaviruses, and filoviruses, all of which are responsible for causing hemorrhagic fever (Furuta et al., 2017). These specific antiviral profiles may ensure FPV as a promising therapeutic candidate for unremedied RNA viral infections. It is interesting to note that some promising antiviral drugs are under preclinical or clinical-stage and others are already got FDA approval (**Table 2**).

**Table 2:** A list of potential antiviral drugs, which are already FDA approved or under in either preclinical or clinical stage.

Antiviral drug	Feature and application	Current status
Darunavir	An HIV protease inhibitor Used against AIDS and HIV infections	FDA approved
Baricitinib	Janus kinases 1 and 2 (JAK1/2) inhibitor Anti-inflammatory, antineoplastic, and immunomodulating actions.	FDA approved
Remdsivir	Antiviral activity against SARS-CoV and MERS-CoV	Clinical
Favipiravir	Effective RdRp inhibitor Used to treat the infection of influenza virus	Approved/clinical
Camostat mesilate	Trypsin-like protease inhibitor Inhibit the function of epithelial sodium channel	Approved/preclinical
Rivapirin	Alleviate HP-C virus and other RNA virus	FDA approved
Chloroquine diphosphate	Toll-like receptors and autophagy inhibitor An aminoquinoline antimalarial	FDA approved
Nitazoxanide	Antiprotozoal actions	FDA approved

### Immune Enhancing Agents

#### Interferons

Interferons (IFNs) are a major category of cytokines, which are a part of our innate immune system. IFNs can be divided into two major groups: type I and type II. IFN-α is a type-I IFN and it had been successfully produced outside the body and was able to restrain viral replication *in* human and animal models (Kint et al., 2015). Type-I IFN, namely, IFN-β had been successfully tested *in vitro* to treat SARS-CoV infection, while IFN-γ does not have any activity against the replication of SARS-CoV (Chen et al., 2004; Morgenstern et al., 2005). The mechanisms behind anti-coronaviridae activity lie in the ability of IFNs to regenerate the propensity of the innate immune response against the virus, according to Kuri et al. (2009). Further investigations by Tan et al. (2004) suggested that cytopathogenic effects or viral alteration of cell structure can be reversed by specific IFN-α and β subtypes such as αn1, α3, β1b, and human leukocyte IFN-α while given in cell culture. Specific and targeted IFNs such as PEGylated recombinant IFN-α2b, generally used against HP-C infections, can protect specific types of alveolar cells in macaques infected with SARS (Haagmans et al., 2004). This targeted therapeutics had been proved to have preventive effects in the lungs if administered 3 days earlier the infection (Bijlenga, 2005).

The recombinant IFN-α2b was used in a pilot clinical study and it was believed to be a potential treatment option for SARS (Loutfy et al., 2003). During the MERS-CoV pandemic, recombinant IFN-α2a in combination with riboflavin was successfully reduced the virus load and many patients could be effectively treated (Mustafa et al., 2018). Such positive outcomes of different types of IFNs support for its use against SARS-nCoV.

#### Intravenous γ-Globulin

It was reported that the best safety profile for immunomodulation to date can be achieved by intravenous administration of γ-globulin (IVIG) (Zhang and Liu, 2020). In Singapore, IVIG was widely used in the SARS epidemic in 2003, one-third of patients got some adverse reactions like thrombo-embolism resulting in blockage of artery of the lungs. Even prophylactic use of low molecular weight heparin could not prevent the adverse reaction of IVIG (Lew et al., 2003). Hypercoagulation and hypofibrinolysis due to IVIG-mediated enhanced viscosity of blood is the main reason for the adverse effects (Dalakas and Clark, 2003). Cao et al. (Li et al.) treated high-dose IVIG on 3 patients who were critically in COVID-19 and they found that high-dose IVIG alleviates the severity of COVID-19. Therefore, randomized studies should be continued at high-dose IVIG for the mitigation of COVID-19.

#### Tocilizumab

Tocilizumab (TCZ) plays a crucial role as a recombinant monoclonal antibody in the human body and shows their function, through binding to either soluble or membrane-type IL-6 receptor, as IL-6 receptor antagonist (Giamarellos-Bourboulis et al., 2020). It has already confirmed that the upregulation of inflammatory cytokines including IL-6 is observed in patients who suffer from COVID (Messina et al., 2020). Several clinical trials demonstrate that TCZ is very effective in deteriorating the pathogenesis of COVID-19. A study in China on 21 patients with severe COVID-19 showed that TCZ causes absorption of pulmonary lesions in about 90.5% infected patients, while C‐reactive protein (CRP) values, body temperature, and the ratio of peripheral blood lymphocytes bring back to normal condition. Of note, no adverse effects are found after using TCZ (Xu X. et al., 2020). Di Giambenedetto et al. (2020) reported in their study that 3 COVID-19 infected patients had a severe respiratory infection but TCZ use significantly declined respiratory symptoms, reduce fever, and decrease CRP values; no adverse events were noticed. Furthermore, a retrospective study was performed on 15 COVID-19 infected patients (mean age 73 years) in Wuhan, China. A group of researchers treats TCZ on COVID-19 patients as a therapeutic candidate and after treating TCZ, IL6 levels were spiked shortly and then significantly reduced the overexpression of IL-6 (Luo et al., 2020). Noteworthy, a positive effect of TCZ is only obtained against critically ill patients after repeated doses and there is no side effect from TCZ (Luo et al., 2020).

#### Thymosin α-1

**Figure 5:**
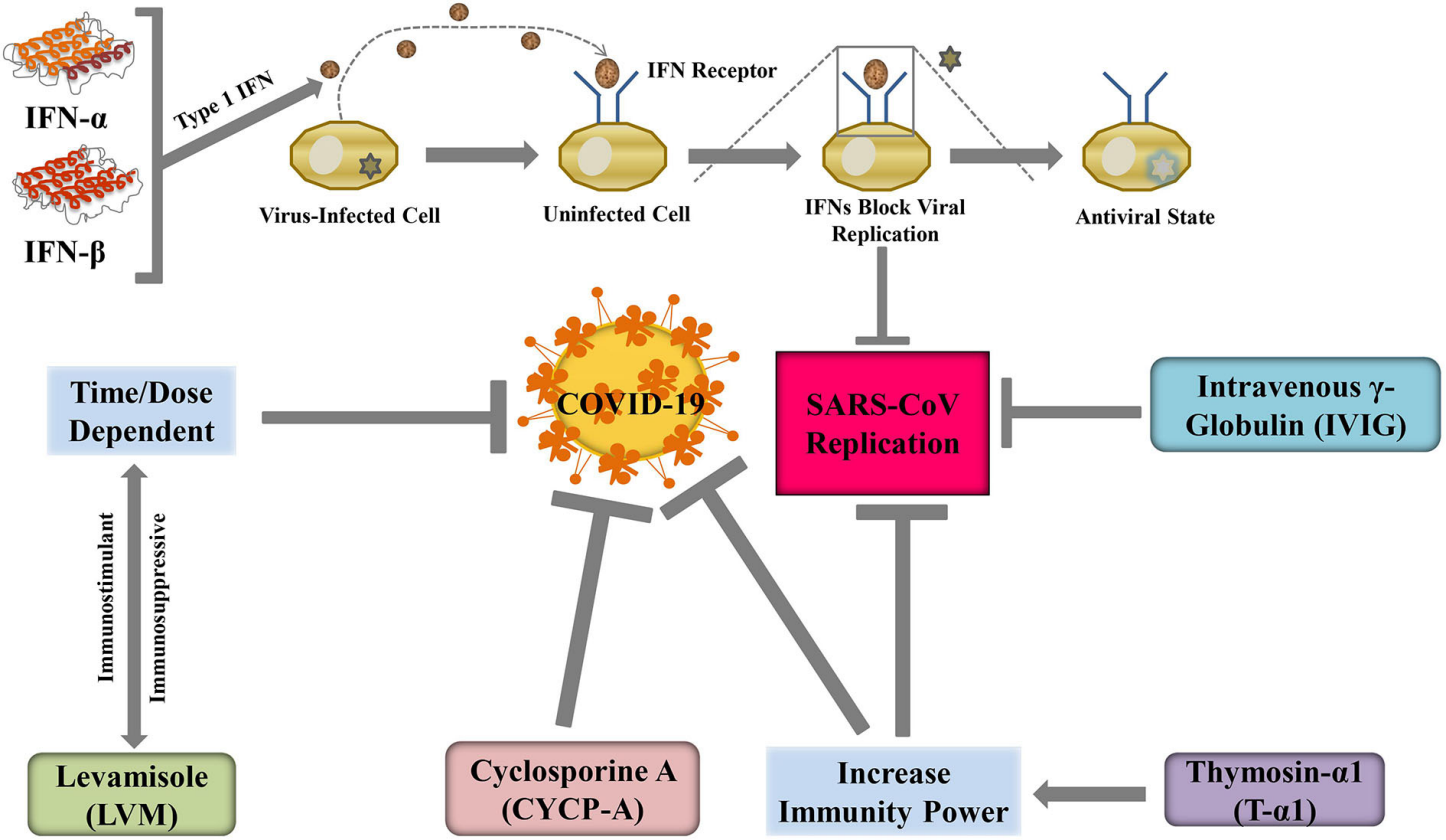
Immune enhancing agents may suppress COVID-19 severity through regulating SARS-CoV replication.

The compensated immune system can be restored to normal function and regulation by another peptide molecule named thymosin α-1 (Tα-1) (Matteucci et al., 2017). Soon after it first came to literature in 1966, it became famous for its capability for stimulating the immune response of the body (Costantini et al., 2019). Later for patients with a hindered immune system, it was commercially synthesized and prescribed (Pica et al., 2018). There are several mechanisms of Tα-1 actions, primarily it can facilitate the development of lymphocytes in the thymus (thymocytes) and prevent the death of those cells by enhancing glucocorticoid resistance (Baumann et al., 1997). During the 2003 SARS epidemic, Tα-1 showed significant activity and played a pivotal role in controlling the epidemic situation (**Figure 5**) (Kumar et al., 2013). During the current pandemic of COVID-19, the administration of methylprednisolone in patients is associated with a reduced number of thymocytes due to glucocorticoids. Concurrent administration of Tα-1 may be able to prevent this side-effect of methylprednisolone.

#### Cyclosporine A

In transplantation of tissue, suppression of immune response is very important to grow acceptance to foreign tissue. Immunosuppressive agent cyclosporine A (CYCP-A) can be used for this purpose and practically it can help patients to survive by inhibiting graft rejection (Ziaei et al., 2016). As an immunosuppressive agent, CYCP-A can be used in the treatment of the autoimmune disorder. In a study conducted by Luo et al. (Luo et al., 2004), it was observed that nucleocapsid protein (NP) of SARS-CoV had a high affinity to a human protein named cyclophilin A and without NP, assembly of viral parts is not possible. Interestingly, CYCP-A also exerts its activity by binding to cyclophilin A which is also a key mediator for viral infection (Dawar et al., 2017). Cyclophilin A inhibits CoV replication of all genera including the bronchitis virus and SARS-CoV (Pfefferle et al., 2011). So, derivatives of CYCP-A may show similar activity against CoV and such newly synthesized compounds can be considered as possible candidates for the treatment of COVID-19. However, Berth-Jones et al. (2019) has revealed that CYCP slightly increases the risk of respiratory tract infection. This study also indicates the use of CYCP is significantly increased renal dysfunction and hypertension. So, the use of CYCP is unclear until now. An amazing data obtained in 2013, CYCP has been reported to reduce CoV replication *in vitro* model (de Wilde et al., 2013). Although *in vivo* effect of CYCP-A is not clear yet (Sbidian et al., 2020). As a result, it wouldn’t be a good decision to use CYCP-A in COVID-19 infected patients. Another study recommends that CYCP-A can be used as a dose of 1 mg/kg/day or below (Rademaker et al., 2020).

#### Levamisole

There is a new immunity-boosting low-molecular-weight compound named levamisole (LVM) which enhances the activity of cellular immunity in control, healthy laboratory animals (Renoux, 1980). Conversely, depending on the dose and dosage regimen, LVM can either stimulate or suppress immunity power in a patient. As a result, its clinical trials were performed carefully. Joffe et al. revealed that ascorbic acid could alter immune suppressive actions when given concomitantly with LVM (Joffe et al., 1983). LVM increases lymphocytes and boosts the immunity power of the body (Ludvigsson, 2020). Besides, LVM can easily bind with Papaine Like Protease (PL-pro) of the virus which may decrease the severity of COVID-19 (Oladele et al., 2012). This drug can also reduce the expression of TNF α and IL-6, which are responsible for COVID-19 pathogenesis. Furthermore, a randomized, open-labeled, phase-III clinical trial (NCT04360122) on LVM is under investigating in 100 older healthy healthcare workers who are randomly divided into four groups of 25 each to take either LVM (150 mg/day for 2 days per week), Isoprinosine (1 g 3 times per day daily), combined LVM and isoprinosine, or no-intervention for 2 months to determine the effect of LVM and Isoprinosine as immune-prophylaxis on the occurrence of COVID-19 infection. Thus, there is a possibility of LVM to be utilized as an anti-COVID-19 agent.

### Vitamins and Omega-3-Fatty Acids

#### Vitamin A

Vitamin A is referred to as a first fat-soluble vitamin where beta-carotene is its precursor. Three active forms of vitamin A including retinol, retinal, and retinoic acid are present in the human body. Vitamin A is also known as anti-infective vitamin and researchers believe that deficiency of particular nutritional elements is responsible for the impairment of immune response (Kańtoch et al., 2002). Measles and diarrhea can be occurred due to the deficiency of Vitamin A (Semba, 1999). Noteworthy, the severity of measles is exhibited in children who have affected in vitamin A deficiency (Villamor et al., 2002). However, Semba et al. (Semba, 1999) revealed that vitamin A alleviated the morbidity and mortality of various infectious diseases, for example, measles, measles-related pneumonia, diarrheal disease, malaria, and HIV infection. Furthermore, vitamin A also alleviates life-threatening infectious disorders, including lung disease, HIV, and malaria. Infectious bronchitis virus (IBV) is one kind of CoV and the infectious effect of this virus is more severe in vitamin A deficient model compared with adequate vitamin A model (Trottier et al., 2009). Viral replication is also inhibited by vitamin A and retinoids. Vitamin A deficiency is generally associated with many respiratory dysfunctions. This happens because vitamin A helps in the development of lung muscles (Timoneda et al., 2018). COVID-19 holds its main effects on the respiratory system. In this context, treatment with vitamin A is completely appropriate (Siddiqi and Mehra, 2020). It has already been clinically proved that vitamin A can help patients to show some level of resistance to the disease (Villamor and Fawzi, 2005). However, a study on elderly patients conferred that vitamin intake cannot significantly assist to improve the efficacy of a vaccine. More specifically, the effects of vaccine were not affected by zinc, β-carotene, α-tocopherol, and retinal (Gardner et al., 2000). Lower respiratory diseases (LRD) are seldom affected by supplementation therapy such as vitamin A. This fact is proved in a separate study with children (Chen et al., 2008). So, in the treatment of COVID-19 associated LRD, vitamin A administration has less potential than previously thought.

#### B Vitamins

B vitamins are water-soluble and act as coenzymes. Each vitamin B has a unique role. For example, the energy metabolism of all cells is regulated by vitamin B_2_ (riboflavin). The deficiency of vitamin B_2_ was suspected to occur among the US elderly. Keil et al. (Bates et al., 1982) have recently reported that in human vitamin B_2,_ plasma products, and exposure to UV light, decrease MERS-CoV titer effectively (Keil et al., 2016). In both prophylactic and therapeutical settings, vitamin B_3_, also known as nicotinamide, could enhance the death of Staphylococcus aureus by a myeloid-specific transcription factor (Kyme et al., 2012). Additionally, vitamin B_3_ treatment showed a strong anti-inflammatory effect whereby neutrophil infiltration into the lung was substantially inhibited during ventilator-induced lung injury. However, the production of essential hypoxemia was also contradictory (Jones et al., 2015). Vitamin B_6_ is also required in protein metabolism, participates in many body-tissue reactions, and also plays a key role in immune systems. Deficiency of vitamin B suppresses the immune response of the host, so they can be given to virus-infected patients to boost up their immune system. Furthermore, oxidative stress is common in COVID-19 patients. It can be determined by a protein marker called homocysteine. In a short-term (6 month) randomized controlled trial (RCT), it had been shown that multivitamin therapy, predominantly with vitamin B complexes, can alleviate the increased level of plasma homocysteine (Ford et al., 2018). Similar results were observed in the long term RCT of 7 years. A complex of folic acid and other B-group vitamins were tested in 300 women and tested against placebo therapy. Cardiovascular disease markers were controlled to the normal level but markers corresponding to vasculitis remained unchanged (Christen et al., 2018). However, more studies are required with a more fitting design corresponding to COVID-19 with a big sample size. Only after those epidemiological studies, it would be appropriate to comment if vitamin B complexes can assist the immune system to protect against COVID-19.

#### Vitamin C

Vitamin C is another vitamin that is water-soluble and is sometimes known as ascorbic acid. Vitamin C, which is widely used as an antioxidant, and plays an important role in collagen synthesis in connective tissues. It also enhances immune function and protects against CoV infection (Hemilä, 2003). For example, Atherton et al. (1978) stated that vitamin C improved resistance to avian CoV infection of chicken embryo tracheal organ cultures. Vitamin C can also be used as a mild antihistamine to relieve symptoms including cough, sneezing or stuffy nose, swollen sinuses, and snow (Field et al., 2002). Three human-controlled trials have shown that vitamin C supplemented groups have a substantially lower incidence of pneumonia, indicating that vitamin C may prevent inflammation of the respiratory tract under some conditions (Hemilä, 1997). In common cold, influenza, and pneumonia, a low dose of vitamin C or L-ascorbic acid is generally suggested as an additional preventive measure (Hemilä, 2017). Indeed, the efficacy of vitamin C in lowering the duration of common cold symptoms had been proved by interventional studies (Hemilä, 2017). Vitamin C administration had been found effective in mice with H1N1 influenza and additional stress (Patel et al., 2017). In those mice, some internal antiviral pathways are shown to be activated such as antiviral signaling in mitochondria and interferon regulatory factor 3. Vitamin C therapy also gave experimental mammals control over steroid hydroxylating enzymes and NF-κB expression (Patel et al., 2017). However, only a few controlled trials proved the efficiency of L-ascorbic acid (Hemilä and Louhiala, 2013). For instance, a dose of 200 mg per day of vitamin C is showed fruitful in a study. The trial was carried out in elderly patients for 4 weeks. The pulmonary conditions of a significant number of patients were improved after the treatment (Hunt et al., 1984). Similar results were observed in other studies as suggested by a meta-analysis of nine RCTs. The meta-analysis summarized that 700–800 mg of vitamin C supplementation per day can relieve symptoms of the common cold in a short period (Ran et al., 2018). However, a similar meta-analysis involving eight RCTs has showed that vitamin C is unable to prevent upper pulmonary tract infections in children (n = 3,135) in doses up to 2 g/day. Still, the duration of the disease could be reduced by 1.6 days upon treatment (Vorilhon et al., 2019). So, in COVID-19 induced lower respiratory tract infection, vitamin C could be an effective therapeutic option.

#### Vitamin D

The main source of vitamin D in the diet through eggs, fish, mushrooms, and fortified milk, and it is well-known for regulating the immune system (Mosekilde, 2005). Vitamin D is not only a vitamin but also a hormone that can be synthesized in the presence of sunlight within our body. It also promotes the maturation of various cells, including immune cells, besides its role in maintaining bone integrity. A large number of healthy adults with low vitamin D levels have been recorded, mostly in late winter (Tangpricha et al., 2002). Further, individuals with housebound and institutionalized working at night, like other seniors who experience minimal access to sunlight, may be deficient in vitamin D (Holick, 2004). In winter 2019, COVID-19 was first reported and primarily affected elderly people in the middle ages. Vitamin D might not be appropriate for virus-infected citizens. However, the decreased status of vitamin D in calves was found to cause bovine CoV infection (Nonnecke et al., 2014). According to human clinical trials, the mixed effects of vitamin D have been observed. Zhou et al. (2018) has reported that the defensive effect of vitamin D intake concerning prevalence and the severity of influenza in China from low- to high-dose vitamin D in infants. A recent study has also demonstrated mixed results in intervention trials (Gruber-Bzura, 2018). Vitamin D therapy, in a meta-analysis administration of vitamin D, enhanced conditions in patients with chronic obstructive pulmonary disease (COPD), although this was not triggered merely by infection (Li X. et al., 2020). In a study by Grant et al. (2020) demonstrated that intake of vitamin D decreased the risk of COVID-19 and influenza infections and mortality owing to related inflammatory condition and anti-microbial peptides including defensins and cathelicidin and by controlling adaptive immunity including decreasing the responses of Th1 helper cell. Recently, Raharusun et al. has reported that most of the death cases of SARS-CoV-2 infection are older and male and have the pre-existing state and under usual serum level of vitamin D (Subscriptions et al., 2020). Therefore, vitamin D might be a potential treatment option for this new virus.

#### Vitamin E

Vitamin E, a lipid-soluble vitamin, plays a pivotal role as a free radical scavenger and reduces oxidative stress (Galmés et al., 2018). Vitamin E deficiency has been reported to increase myocardial injury and intensify coxsackievirus B3 infection in mice model (Thevarajan et al., 2020). Vitamin E deficiency in claves is associated with bovine CoV infection (Nonnecke et al., 2014). Vitamin E supplementation in individuals appears to restore the generation of IL-2, which enhances the proliferation of T-cell and boosting the immune system (Meydani et al., 2005; Lewis et al., 2019). According to the study of Ghani et al. (2019) the supplementation of tocopherol or tocotrienol revealed increased expression of various genes connected with the immune response in Malaysian adults volunteers. Remarkably, the particular genes changed between the two groups were dissimilar. Moreover, the intake of vitamin E, after a first hospitalization in the elderly with pneumonia, was associated with decreased 63% re-hospitalization within 90 days followed by the first hospitalization in aged patients with pneumonia (Neupane et al., 2010). Meydani et al. (Meydani, 1997) conducted a RCT in healthy, elderly participants taking 200 mg/d of vitamin E capsules for 4 months and had been found that a 65% increase in delayed-type hypersensitivity skin response and a significant increase in antibody titer to tetanus vaccine and hepatitis B in comparison with placebo, emphasizing involvements in T-cell induced functions. Besides, it has also been observed that the administration of 50 mg/d vitamin E among 2216 smokers for 5–8 years decreased pneumonia prevalence by 69% in older people (Hemilä, 2016). Thus it can be considered that Vitamin E might be an effective therapeutic choice for COVID-19.

#### Omega-3 Polyunsaturated Fatty Acids

Omega-3 polyunsaturated fatty acids (ω-3 PUFAs) are important mediators and regulate diverse inflammatory cytokines and chemokines (Parolini, 2019; Radzikowska et al., 2019). During viral infection, ω-3 PUFAs show its anti-inflammatory functions and reduce inflammation. Sharma et al. (2013) have revealed that the administration of ω-3 PUFAs alleviates the severity of acute phenomena through increasing the immune response. Furthermore, ω-3 PUFAs can reduce the expression of ERK1/2 MAPK, NF-κB, and COX-2 (Calder, 2013). Additionally, oral administration of ω-3 PUFAs inhibits ROS generation and downregulates the level of TNF-α, IL-1β, IL-6, and IL-8 during viral infection (Sorensen et al., 2014). Long-chain PUFAs are important inflammatory and adaptive immune reaction mediators (Cai et al., 2018) ω-3 and ω-6 PUFAs are primarily inflammatory and pro-inflammatory. These are precursors of resolvins/protectins and prostaglandins/leukotrienes respectively (Cai et al., 2018). Bégin et al. (1989) found a specific lack of ω-3 long-chain PUFA among the lipids in patients with AIDS which are abundant in fish oils. Protectin D1, the lipid mediator derived from ω-3 PUFA, may also significantly reduce the replication of the influenza virus through RNA exporting machinery. A recent report showed that the combination of protein D1 with peramivir reduced the mortality rate of mice after affecting the flu (Morita et al., 2013). Leu et al. (2004) found that many PUFAs had anti-HCV activities.

Supplementation of omega-3 was used earlier in ARDS. It has been observed that the enteral administration of fish oil (rich in antioxidants and ω-3 PUFAs) can enhance oxygenation and clinical benefits in the patients of the intensive care unit (ICU) (Singer and Shapiro, 2009). In a study by Li et al. (2015) suggested a favorable effect merely for patients with ARDS, after conducting a systematic review in 2015. Recently, a meta-analysis emphasized the significance of clinical trials to elucidate the use of antioxidants and ω-3 fatty acids in patients suffering from ARDS to determine the favorable effects to decrease the periods of ICU stay and the total number of days passed on ventilators (Dushianthan et al., 2019). Though the effect of ω-3 administration in ARDS has to be better elucidated, however, it plays a crucial role in decreasing reactive oxygen species and pro-inflammatory cytokines, including IL-6, IL-8, IL-1β, and TNF-α (Langlois et al., 2019). Therefore, ω-3 may be considered as one of the potential antiviral treatments for COVID-19.

### Oligoelements and Immune Response

#### Selenium

Selenium (Se) is an important trace element for redox mammalian biology (Rayman, 2012). The host’s nutritional status is very important in the prevention of infectious diseases (Beck and Matthews, 2000). Nutritional deficiency affects both the immune response of the host and the viral pathogens themselves (Guillin et al., 2019). Dietary Se deficiency causing oxidative stress will change the genome of the host such that the host may become much more virulent with a usually benign or mildly pathogenic virus (Guillin et al., 2019). Se deficiency also causes not only host immune system defects but also a rapid mutation of benign RNA virus variants into virulence (Harthill, 2011). Beck et al. (2001) reported that a deficiency of Se can aggravate influenza virus infections and also activates coxsackievirus genome, changes allowing an avirulent virus to develop virulence after genetic mutations (Beck et al., 1995). The Se may help a group of enzymes to prevent the formation and oxidative damage of cells and tissues in conjunction with vitamin E (Harthill, 2011). The synergistic effect of Se with ginseng stem-leaf saponins has been reported to induce an immune response to live infectious bronchitis CoV (Ma et al., 2019). Recently, Mahmoodpoor et al. conducted an RCT in critically ill patients suffering from ARDS took sodium selenite (a form of Se) (Mahmoodpoor et al., 2019). In this study, the concentrations of Se are linearly connected with serum levels of GPx, antioxidant activity, and ferric reducing antioxidant power. In contrast, both IL-6 and IL-1β serum concentrations are inversely connected with serum levels of Se. Besides vitamins, Se could also be a favorable therapeutic agent for treating the COVID-19.

#### Zinc

Zinc (Zn) is one of the most researchable trace elements because of considering its effect on the host immune system (Kabir et al., 2020b). Deficiencies in Zn are classified as acute, chronic, and subacute deficiency. Subacute deficiency affected about 4 million people in the USA and it was considered as the most common around the world (Walsh et al., 1994). It has been reported that Zn deficiency elevates the susceptibility of infectious disease and decreases thymus size and depletes macrophages and lymphocytes in the spleen in Zn deficient mouse model (Fraker et al., 1986; Haase and Rink, 2009). Besides, suboptimal Zn is also associated with the reduction of T-cell function resulting in inhibition of antibody response (Kruse-Jarres, 1989). Walsh et al. (1994) revealed that immune status was restored when Zn status was normal. However, an excessive level of Zn contributes to reducing polymorphonuclear leukocytes activities, antibody production, and T-cell proliferation (Schlesinger et al., 1993). These data demonstrate that immune system response, including stimulation and suppression, depends on the concentration of Zn. Further studies are required to examine the proper role of Zn for determining immune system functioning.

Zn is a trace dietary mineral and is essential to sustain and improve both the innate and adaptive immune systems (Maares and Haase, 2016). Zn deficiency results in humoral and cell-mediated immunity dysfunction and facilitates susceptibility to infectious diseases (Tuerk and Fazel, 2009). Administration of Zn to children with Zn deficiency may reduce the morbidity and mortality caused by lower airway infections (Awotiwon et al., 2017). An increase in intracellular Zn concentration with Zn ionophores such as pyrithione will effectively impair a variety of RNA viruses replication (te Velthuis et al., 2010). Furthermore, Zn and pyrithione combinations at low concentrations inhibit SARS-CoV replication (te Velthuis et al., 2010). A recent study has demonstrated that low levels of Zn in older people and its association with pneumonia has been highlighted (Barnett et al., 2010). The rate of mortality because of pneumonia has also been claimed to be twice as high in patients with low levels of Zn versus patients with standard Zn status (Barnett et al., 2010). Furthermore, Zn has also been reported to enhance the symptoms of the common cold. In a study by Mossad et al., (1996**)** conducted an RCT in 100 patients with the symptoms of the common cold received Zn (13.3 mg) until symptoms were present. In this study, Zn considerably decreased the length (from 7.6 to 4.4 days) of symptoms of the common cold in comparison with a placebo. Zn supplements can, therefore, be used to reduce symptoms associated with viral infections such as diarrhea and infections with lower respiratory tract and might be effective against COVID-19 itself.

#### Copper

Copper (Cu) is another important nutrient, which maintains the proper mechanism of the human body. Cu-deficient models are more susceptible to bacterial, parasitic, and viral infections (Stabel et al., 1993; Djoko et al., 2015). Menkes syndrome is occurred due to malabsorption of Cu and this syndrome results in infectious bronchopneumonia (Prohaska and Lukasewycz, 1990a). Animal studies revealed that Cu-deficient mice reduced B-cell activity, antibody responses to a variety of antigens, and impairs plaque formation (Prohaska and Lukasewycz, 1981). Besides, the proliferative responses of T-cell to several antigens are lower in Cu-deficient animals than Cu-sufficient animals (Prohaska and Lukasewycz, 1990). Natural killer cell response is reduced and lymphocyte subset populations are changed in Cu-deficient animals. Furthermore, the number of B-cells elevated in low Cu diet humans but total T-cells or T-cell subsets numbers did not any significant change (Water, 2000). Sufficient Cu supplementations are essential for immune function because it has a wide range of biochemical activities as a cytochrome c oxidase (CYTC-ase), the cofactor for ferroxidase (COF-ase), and Zn-Cu superoxide dismutase (Zn-CuS-ase), resulting in inhibition of oxidative stress (Water, 2000). Therefore, adequate Cu treatment might be a necessary key for maintaining immune functions.

#### Iron

Iron is essential not only for the host but also for the pathogen. Iron deficiency is associated with impairment of host immunity, while the excessive level of iron can cause oxidative stress resulting in harmful viral mutations (Wessling-Resnick, 2018). In addition, iron deficiency is also responsible for elevating the infection of the recurrent acute respiratory tract (Jayaweera et al., 2019). It is well-known that iron has a positive effect on bacterial infections (Cherayil, 2011) and viral infections (Drakesmith and Prentice, 2008), such as respiratory infections (Ali et al., 2017), suggesting that the homeostasis and levels of iron are strongly controlled. Furthermore, the absorption of iron is downregulated through hepcidin (Cherayil, 2011) during inflammation to limit the available supply of iron for increasing virus and bacteria particles rapidly and to regulate excessive oxidative stress. In contrast, Dhur et al. (1990) revealed that the production of antibodies was reduced in mice exposed to the influenza virus due to long periods of iron deficiency. This type of effect has also been observed in elderly adults, linking the deficiency of iron to disrupted cell-mediated and innate immunity (Kemahli et al., 1988). Recently, Jayaweera et al. (2019) has performed a case-control study in hospitalized children who are 2–5 years old taking iron supplementation for 3 months, reappearances of urinary tract infections, gastroenteritis, and acute respiratory tract infections are considerably decreased. Therefore, iron may also be a favorable therapeutic candidate for treating the COVID-19.

### Specific Treatments

#### Chloroquine Phosphate

Chloroquine phosphate (CLP) is used as a potent drug for the remedy and prevention of malaria. In *in vitro* studies, at low micromolar concentration, CLP was found to inhibit COVID-19 infection, where half-maximal effective concentration (EC50) and half-cytotoxic concentration (CC50) were 1.13 μM and greater than 100 μM, respectively (Wang M. et al., 2020). Several clinical trials have been conducted in Wuhan, China to measure the safety and efficacy of chloroquine or hydroxychloroquine for the treatment of pandemic COVID-19. After treating CLP on COVID-19 patients, the investigators ensured that CLP is superior finding in inhibiting pneumonia, developing virus-negative conversion, improving lung imaging, and limiting disease course from chines news briefing (Yan et al., 2013). There is no noticeable severe adverse effect after treating CLP on COVID-19 infected patients. Based on these findings, a conference was organized on February 15, 2020 by Chinese regulatory authorities and clinical trial organizers, and they revealed that CLP has a potent effect against COVID-19, and this drug is primarily recommended for generating the next version remedy for COVID-19 (Gao et al., 2020). Recently, Huang M. et al. (2020) have performed an RCT in 22 SARS-CoV-2 patients received 500 mg of chloroquine in one group and another group took 400/100 mg of lopinavir/ritonavir respectively for 10 days and observed for a total of 14 days. Furthermore, patients who are treated with chloroquine are more possible to become negative for viral RNA. Indeed, all the chloroquine treated patients turned negative for viral RNA test by day 13. Serious adverse effects were not claimed in these treatment groups. On the other hand, cardiovascular effects, such as hypotension, arrhythmias, decreased myocardial performance, and vasodilation has been claimed after the administration of chloroquine at high doses (Ben-Zvi et al., 2012). Therefore, it is essential to be attentive to the probable risk of chloroquine and hydroxychloroquine when taking at high doses.

#### Ivermectin

Ivermectin (IVM), an FDA approved anti-parasitic agent, shows antiviral effect against a wide range of viruses *in vitro* model (Wagstaff et al., 2012; Wang M. et al., 2020). Caly and colleagues (Caly et al., 2020) determine the antiviral activity of IVM towards SARS-CoV2. They treated 5 mM IVM in SARS-CoV2 infected Vero/hSLAM cell and analyzed SARS-CoV2 replication by RT-PCR. By 24 hours, 93% of viral RNA was reduced from IVM treated samples in comparison with vehicle DMSO. At 48 h, this effect developed to ~5000 fold viral RNA reduction in IVM treated samples compared to the control group. These results indicate that IVM treatment significantly reduced viral material by 48 hours and there is no toxicity observed due to IVM treatment at any time point (Caly et al., 2020). So, taken together their results demonstrate that IVM exerts antiviral actions against SARS-COV2 and inhibits viral replication at a single dose within 24 to 48 h.

#### Coronaviral Protease Inhibitors

Proteases are enzymes that can synthesize new peptide sequences by breaking proteins into smaller polypeptides. In the replication of CoV, two known proteases such as chymotrypsin-like protease (CTLpro) and papain-like protease (PLpro) are important mediators. These enzymes are also known to suppress innate defense systems of the human. So, agents that can inhibit CTLpro and PLpro can be used as anti-viral drugs against COVID-19 (Park et al., 2012).

##### Chymotrypsin-Like Inhibitors

To inhibit CTLpro encoded in COVID-19, a recognized serotonin receptor blocker cinanserin (CIN), can be used. It was proved to be effective against SARS-CoV in recent studies (Chen et al., 2005). The CTLpro had has also been experienced to be encoded in COVID-19 (Chen Y. et al., 2020). Thus, to treat SARS-CoV2, CIN can be very much effective. In addition, natural products like flavonoids, flavonols, flavones, isoflavones, and chalcones, possesses antioxidant and a certain anti-viral activity (Panche et al., 2016). In a study, successful treatment of the HP-C virus was achieved by using a flavonoid extract obtained from pterogyne plant (*Pterogynenitens)* (Shimizu et al., 2017). Another study proved the ability of some flavonoids like herbacetin, rhoifolin, and pectolinarin blocks CTLpro which may contribute to inhibit the progression of CoV (Jo et al., 2020). In the case of MERS-CoV, herbacetin, quercetin 3-β-d-glucoside, isobavachalcone, and helichrysetin were fruitful for similar mechanisms of action (Jo et al., 2019). Another report showed the effectiveness of a conifer-extracted (*Torreyanucifera*) biflavanoid to antagonize CTLpro of SARS-CoV (Ryu et al., 2010).

##### Papain-Like Protease Inhibitors

As a deubiquitinase encoded by novel CoV, papain-like protease (PLpro) blocks the interferon activity of the host against viruses. Several agents can be designed to inhibit PLpro. For example, diarylheptanoids (DIH) extracted from the barks of Japanese Alder (*Alnus japonica*) can inhibit the PLpro found in SARS-CoV (Park et al., 2012). So, combination therapy of CIN/flavonoids with DIH can be selected to inhibit both CTLpro and PLpro at a time against COVID-19.

#### ACE2 Inhibitors

The first homolog of ACE found in *Homo sapiens*. As a carboxypeptidase, ACE2 hydrolyzes the peptide bonds of specific polypeptides (Warner et al., 2004). More specifically, ACE2 converts a human polypeptide hormone angiotensin (ANG) II known for its vasoconstrictor properties and thus mediates several conditions related to cardiac pathology. Due to its enzymatic activity, ANG II is converted to ANG I-VII (Warner et al., 2004). However, the spike (S) protein on the envelope of the SARS-CoV shows an affinity towards ACE2 and uses this enzyme as a way of entering the human cell (Dimitrov, 2003; Li et al., 2003). More elaborately speaking, using S glycoprotein, CoV can fuse with the cell membrane after binding to ACE2 which acts as a host cell receptor (Simmons et al., 2004). Moreover, ACE2 is used as a sole receptor for the entry of COVID-19, although other CoV receptors, including aminopeptidase N and dipeptidyl peptidase, are not used for the entry. Consequently, the increased expression of ACE2 may facilitate COVID-19 infection in diabetes and hypertensive patients, and thus increased the risk of developing fatal COVID-19 (Li et al., 2017). Therefore, patients should be carefully monitored while using ACE2-stimulating drugs such as ACE inhibitors or ARBs. As part of the ongoing monitoring of the safety of medicines, recently published studies on the use of ACE inhibitors and ARBs during the COVID-19 pandemic were reviewed and showed that these concerns are not supported by the latest clinical evidence (Zhang P. et al., 2020). Currently, a clinical trial is going on to verify whether the chronic intake of RAS inhibitors modifies the prevalence and severity of the clinical manifestation of COVID-19 (ClinicalTrials.gov, 2020a). So, ACE2 antagonists hold a great future for treating COVID-19 (Yeung et al., 2006).

##### Chloroquine and Emodin

The world-famous anti-malarial drug chloroquine had been in use since 1934 for its anti-protozoal properties. However, its anti-viral properties are also discovered (Savarino et al., 2003). More specifically, it can obstruct the binding of spike protein with ACE2, thus prevents the SARS-CoV components from entering the host cell (Vincent et al., 2005). FDA recommended the emergency use authorization (EUA) of hydroxychloroquine or chloroquine to treat certain hospitalized patients with COVID-19. However, based on ongoing large RCTs and emerging scientific data indicated that the medicines have no benefit of recovery or mortality and that the suggested doses are unlikely to inhibit viruses causing COVID-19 (FDA, 2020). Rather, serious heart rhythm problems and major organ problems or failure were reported. Further, FDA announced cautions against the use of hydroxychloroquine or chloroquine for COVID-19 outside of the hospital setting or a clinical trial due to the risk of heart rhythm problems. Currently, a large scale clinical trial is ongoing to check the therapeutic effects of chloroquine phosphate in COVID-19 infected patients (ClinicalTrials.gov, 2020c). In contrast, an anthraquinone named emodin (EMD) extracted from Rhubarb (Rheum) and knotweed (Polygonum) shows extensive anti-viral efficacy (Alves et al., 2004). EMD can be a potential agent against COVID-19 as it does not allow the S-glycoprotein to bind with ACE2 (Ho et al., 2007).

##### Promazine and Nicotianamine

A phenothiazine derivative and an anti-psychotic agent promazine (PRZ) had also been found effective in blocking the replication of SARS-CoV (Zhang and Yap, 2004). It has a similar mechanism of action like EMD and can significantly block ACE-2 and S-protein contact and binding (Ho et al., 2007). So, treatment against COVID-19 can be done through inhibition of ACE2 receptor protein and human mAb like scFv80R, chloroquine, EMD, and PRZ can be used in response to the current pandemic. On the contrary, nicotianamine (NTA) is a metal chelating agent found in soybean seed and other plants (Trampczynska et al., 2006). This agent is one of the newest agents that are found to block ACE2, and thus, it is another option for the treatment of COVID-19 (Takahashi et al., 2015). A phase III clinical trial is running to the standard therapeutic protocol in COVID-19 patients hospitalized for respiratory symptom management (ClinicalTrials.gov, 2020b).

### Anticoagulant Treatment

In many cases, anticoagulant therapy such as heparin is effective against severe COVID-19. An investigation by Tang et al. (2020a) demonstrated that severe COVID-19 patients develop elevated platelet levels that can cause multiple complexities. Those researchers showed patients with severe COVID-19 benefits from low molecular weight heparin therapy (Tang et al., 2020a). Yin et al. demonstrated that the positive effect of anticoagulants is only limited to patients with severe COVID-19 associated pneumonia (Yin et al., 2020). A probable reason for the effectiveness of heparin depends on its interaction with the virus itself. A portion of heparin can bind to the SARS-CoV2 S1 receptor binding domain of the COVID-19 virus or SARS-CoV2 S1 RBD (Mycroft-West et al., 2020). So, there is a potential for future drug development studies based on this molecular interaction of heparin and the virus. In practice, more and more evidence is rowing for the use of anticoagulants in severe COVID-19 cases. A study in Spain with 2,075 patients showed a significant fall of death rate (adjusted hazard ration = 0.55) when heparin intervention is used (Ayerbe et al., 2020). In China, many patients with sepsis-induced coagulopathy were treated with heparin-based therapy. Experts showed that a significant number of them survived. Still, long term study (28 days) could not confer any correlation between heparin and morbidity (Tang et al., 2020a). Besides, in USA systemic anticoagulants were found effective in patients requiring ventilation (n = 395). A study showed that the mortality rate was decreased from 62.7% to 29.1% among those patients (Ayerbe et al., 2020).

## Conclusion

The transmission of novel COVID-19 is increasing with an unexpected speed from humans to humans around the world. Noticeably, mode of transmission contributes to accelerating the spreading of COVID-19 which may assist a scientist to discover effective therapeutic measures. Till now, no effective vaccine or drug is found to treat COVID-19, thus some effective remedies are recommended as a basic treatment according to their anti-viral properties. In this pandemic situation, scientists from all over the world are trying to discover a preventive vaccine or specific drug that can either lock-down the entry of this virus or inhibit the subsequent replication to alleviate COVID-19. It is interesting to note that some drugs have already passed phase 1 and that several investigational drugs are under clinical trials. Concomitantly, *in silico* studies can be performed to make the drug development procedure rapidly. Noteworthy, anti-viral agents, immune enhancers, basic supplements, and anticoagulant therapy significantly contribute to alleviating SARS-CoV replication. Therefore, these proposed interventions might be promising and compatible therapeutic options for the management of COVID-19.

## Author Contributions

MH and MU conceived the original idea and designed the outlines of the study. MH, SH, AM, SS, IU, and MW wrote the draft of the manuscript. MH, MU, and IU prepared the figures and tables of the manuscript. MU, TB, YB, IB MA, MR, MB-J, SA, SM, LA, and MA-D performed the literature review and improved the manuscript. All authors contributed to the article and approved the submitted version.

## Conflict of Interest

The authors declare that the research was conducted in the absence of any commercial or financial relationships that could be construed as a potential conflict of interest.
